# Supplementary material for: Structural consistency in AI governance: A PMC index assessment with evidence from China’s central-level policies
Source: PLoS One. 2026 Jun 5;21(6):e0337024. doi: 10.1371/journal.pone.0337024 (PMC13240918; doi:10.1371/journal.pone.0337024)
Supplement: S1 Data — S1 File. Full list of the 54 central-level AI policy documents included in the corpus, including policy titles, issuing authorities, and years of issue. S2 File.Text-processing notes and lexical adjustment record used in the word-segmentation stage. S3 File. Detailed indicator scoring matrix for the PMC evaluation system. S4 File. Full PMC results for the five selected representative policies. S5 File. Underlying frequency data used to generate Figure 4. (ZIP) [file pone.0337024.s001.zip › support information/S2_File_Text_Processing_Notes_and_Lexical_Adjustment_Record_English.docx]

**1. Purpose of this file**

This file documents the text-processing procedure and the lexical adjustment strategy used in the word-segmentation stage of the study. It is provided to improve transparency and reproducibility in the text-mining component of the analysis.

**2. Initial segmentation basis**

Chinese word segmentation was conducted in ROSTCM6.0 on the full texts of the 54 central-level AI policy documents included in the corpus. The lexical resource available in the ROSTCM6.0 software environment was used as the initial basis for segmentation.

This lexical resource was not treated as a separately constructed standalone research dictionary. Rather, it served as the initial segmentation support available in the software environment.

**3. Corpus-oriented lexical adjustment**

The study did not rely on the software-environment lexical resource mechanically or without modification. Instead, the segmentation process was further refined in light of the characteristics of the Chinese AI policy corpus.

More specifically, the text-processing stage involved three kinds of corpus-oriented adjustment:

(1) Manual checking of segmentation output.

The initial segmentation results were reviewed against the full policy corpus in order to assess whether segmented expressions aligned with the substantive content of China’s AI policy texts.

(2) Selective retention of AI-relevant policy expressions.

Terms closely related to AI technology, governance, industrial development, public services, and policy implementation were selectively retained where necessary to improve the interpretability of the corpus.

(3) Filtering of structurally repetitive or analytically uninformative expressions.

Expressions such as article numbers, chapter labels, and procedural language were filtered out when they did not contribute to substantive policy analysis.

Accordingly, the segmentation-related lexical support used in this study should be understood as a software-based initial resource that was selectively refined through corpus-oriented manual adjustment.

**4. Analytical role of the lexical resource**

The segmentation-related lexical resource was used only to support text preprocessing, word segmentation, and corpus exploration. It was not used as a direct scoring instrument for the PMC model, nor should it be interpreted as a finalized standalone policy keyword dictionary.

Instead, its role was to improve the alignment between the segmentation output and the substantive vocabulary of China’s AI policy discourse, thereby facilitating subsequent word-frequency analysis and semantic co-occurrence analysis.

**5. Representative examples of AI-relevant lexical entries**

To improve accessibility for English-language readers, representative examples of lexical entries that are substantively relevant to the AI policy corpus are provided below.

| **Related terms** | **Analytical relevance** |
| --- | --- |
| Robots | Core AI technology term |
| Transparency | Governance-related term |
| Automation | Application and industrial term |
| Talent cultivation | Policy support term |
| Demonstration application | Implementation term |
| Independent innovation | Innovation policy term |
| Intelligent healthcare | Application scenario |
| Data security | Governance and regulation term |
| Explainability | AI governance term |
| Government services | Public-service application term |
| Deep learning | Core technical term |
| Cognitive computing | Core technical term |
| Natural human–computer interaction | Core technical term |
| Digital government | Governance term |
| Digital economy | Policy domain term |
| Data elements | Governance and economic term |
| Intellectual property | Policy support term |
| Human–machine collaboration | Technical/application term |
| Brain-inspired intelligence | Frontier AI term |
| Intelligent manufacturing | Industrial application term |
| Public services | Governance and social term |
| Personal privacy | Ethical/governance term |
| Global governance | Macro-governance term |
| Autonomous driving | Application scenario |
| Speech recognition | Core technical term |
| Artificial intelligence | Core AI technology term |

**6. Preprocessing examples of filtered expressions**

Examples of expressions filtered during preprocessing include the following:

| **Type** | **English description** |
| --- | --- |
| Structural markers | Article numbers; chapter labels |
| Procedural expressions | “shall not”; “process”; “should” |
| Repetitive formal expressions | Recurrently repeated formulaic policy language without substantive analytical value |

**7. Note for interpretation**

Because the source corpus consists of Chinese-language policy documents, the original lexical environment and segmentation process are language-specific. The present file provides an English-language description of the segmentation basis, lexical adjustment logic, and representative domain-relevant entries in order to support transparency, reproducibility, and editorial accessibility.
