# Supplementary material for: Structural consistency in AI governance: A PMC index assessment with evidence from China’s central-level policies
Source: PLoS One. 2026 Jun 5;21(6):e0337024. doi: 10.1371/journal.pone.0337024 (PMC13240918; doi:10.1371/journal.pone.0337024)
Supplement: Attachment — Submitted filename: PLos one.docx [file pone.0337024.s003.docx]

**Comparative Evaluation of the Revised Manuscript**

**Manuscript Title:**

**Structural Consistency in AI Governance: A PMC Index Assessment with Evidence from China’s Central-Level Policies**

This comparative evaluation assesses the revised manuscript in relation to the previous reviewers’ comments and the authors’ responses. Overall, the revised version shows substantial improvement in several important areas, including the introduction, literature review, methodological transparency, data-source reporting, interpretation of PMC scores, and discussion section. Most of the previous reviewers’ concerns have been addressed satisfactorily. However, a few minor issues still require attention.

**Previous Reviewer 1**

**Comment 1**

The reviewer requested that the authors improve transparency and reproducibility by providing supplementary materials documenting the policy corpus, text-processing procedures, indicator scoring matrices, and full PMC results.

**Authors’ Revision**

The authors added a new section on transparency and reproducibility and introduced supplementary files S1–S5. They also revised the sections related to data sources, text-mining procedures, variable classification, and indicator scoring.

**Evaluation Outcome**

This concern has been largely addressed. The revised manuscript provides clearer explanations of where the data were obtained, how the policy texts were processed, and how the indicators were operationalized. The addition of a transparency and reproducibility section strengthens the methodological reporting of the study. However, the completeness and adequacy of the supplementary materials should still be verified to ensure that they actually include the full policy corpus, the detailed scoring matrix, and the complete PMC results.

**Comment 2**

The reviewer requested a clearer interpretive framework for PMC scores, including what relatively strong or weak structural consistency means in practical terms.

**Authors’ Revision**

The authors added an explanation of how PMC scores should be interpreted. They clarify that higher PMC scores indicate a more complete, balanced, and internally coordinated policy structure, whereas lower scores indicate weaker coverage or imbalance across policy dimensions.

**Evaluation Outcome**

This concern has been addressed. The revised manuscript no longer presents PMC scores merely as numerical rankings. It now provides a clearer policy-oriented interpretation of the scores and explains how they relate to structural consistency, balance in policy design, and coordination among policy dimensions.

**Comment 3**

The reviewer requested a brief discussion of robustness and potential sensitivity to indicator selection or policy sampling.

**Authors’ Revision**

The authors added a discussion of the potential sensitivity of the results to the indicator system and to the selection of five representative policies. They also clarify that the findings should be interpreted as structurally informative rather than numerically absolute.

**Evaluation Outcome**

This concern has been partially addressed. The authors have discussed robustness and sensitivity at a conceptual level, which is acceptable given that the previous comment requested a brief discussion. However, the manuscript still does not include an empirical robustness test or sensitivity analysis. Therefore, this issue has been addressed at the discussion level, but not fully resolved empirically.

**Previous Reviewer 2**

**Comment 1**

The introduction should engage more deeply with the concept of artificial intelligence governance and define it more clearly and theoretically.

**Authors’ Revision**

The authors added a clearer definition of AI governance and linked it to institutional, strategic, and regulatory arrangements through which the state guides, supports, coordinates, and constrains the development and application of AI.

**Evaluation Outcome**

This concern has been addressed. Compared with the previous version, the revised introduction now provides a clearer conceptual framing of AI governance and connects it more effectively to policy structure, policy instruments, implementation mechanisms, and multi-level coordination.

**Comment 2**

The introduction should not include methodological details.

**Authors’ Revision**

The authors substantially reduced methodological details from the introduction. The revised introduction now focuses more on the research background, theoretical problem, research gap, and overall objective of the study.

**Evaluation Outcome**

This concern has been largely addressed. Although the revised introduction still briefly refers to the corpus and PMC framework, this level of reference is acceptable for introducing the study. It no longer contains excessive methodological detail.

**Comment 3**

The description of the paper’s structure should be removed from the introduction.

**Authors’ Revision**

The section-by-section description of the manuscript that appeared in the original introduction has been removed.

**Evaluation Outcome**

This concern has been addressed. Removing the unnecessary description of the paper’s structure has made the introduction more concise and coherent.

**Comment 4**

The literature review was overly extensive and should be condensed, focusing primarily on AI governance and the PMC Index.

**Authors’ Revision**

The literature review has been reorganized and condensed. It now focuses mainly on two core areas: AI governance and policy, and the PMC Index Model for policy evaluation.

**Evaluation Outcome**

This concern has been addressed. The revised literature review is more focused, more coherent, and better aligned with the main topic of the manuscript. Some further condensation may still be possible, but the main concern has been resolved.

**Comment 5**

The data sources should be reported clearly and transparently, specifying exactly which databases or websites were used.

**Authors’ Revision**

The authors now explicitly identify the main sources used to collect the policy documents, including the Chinese Government Website and PKUlaw. They also report the data retrieval period.

**Evaluation Outcome**

This concern has been addressed. The revised manuscript improves the transparency and traceability of the policy corpus by clearly specifying the data sources and retrieval period.

**Comment 6**

The methodology was not described with sufficient clarity. It was unclear which parts of the texts were used for text mining, what analyses were conducted, what steps were followed, and which tools were used.

**Authors’ Revision**

The authors clarify that the full texts of the 54 policy documents were used as input for text mining. They also describe the analytical sequence, including corpus preparation, Chinese word segmentation, word-frequency analysis, and semantic co-occurrence analysis using ROSTCM6.0 and NetDraw. They further clarify that text mining supported variable construction rather than automatically generating the final PMC scores.

**Evaluation Outcome**

This concern has been addressed. The methodological reporting has improved substantially. The input corpus, text-processing procedures, analytical steps, software tools, and role of text mining in variable construction are now much clearer.

**Comment 7**

The authors were encouraged to refer to Danesh et al. (2021) as a methodological guide for transparent reporting of the text-mining procedure.

**Authors’ Revision**

The authors state in their response that they used the methodological reporting logic of Danesh et al. to revise the text-mining section.

**Evaluation Outcome**

This concern has been partially addressed. The reporting of the text-mining procedure has improved. However, a clear full citation to Danesh et al. (2021) was not identified in the revised manuscript or reference list. If the authors used this study as a methodological reporting guide, the full citation should be added to the text and reference list.

**Comment 8**

It was unclear whether the study relied solely on automated text-mining analysis or also involved researcher interpretation. The analytical basis for Tables 2, 3, and 4 also needed to be explained.

**Authors’ Revision**

The authors now clarify that text mining played a supportive rather than determinative role. They explain that the construction of the indicator system, interpretation of policy elements, and binary scoring were based on structured researcher judgment and explicit coding rules. They also clarify the analytical roles of Tables 2, 3, and 4.

**Evaluation Outcome**

This concern has been addressed. This is one of the most important improvements in the revised manuscript. The distinction between automated text processing and researcher-led interpretation is now clearer, and the analytical basis of Tables 2, 3, and 4 has been better explained.

**Comment 9**

The Discussion section required substantial rewriting. It should focus on the main findings of the study and compare them with previous research rather than presenting descriptive or repetitive content.

**Authors’ Revision**

The authors rewrote the Discussion section and organized it around the principal findings of the study, including the stable developmental-governance core of China’s AI policy system, weaknesses in temporal planning and policy hierarchy, and heterogeneity in incentive arrangements and levels of influence.

**Evaluation Outcome**

This concern has been largely addressed. The revised Discussion section is more analytical and better organized around the main findings. It also links the findings more clearly to previous research. However, the critical comparison with prior studies could still be slightly strengthened.

**Overall Comparative Evaluation**

The revised manuscript has improved substantially compared with the original version. Most of the major concerns raised by the previous reviewers have been addressed satisfactorily. The introduction is conceptually clearer, the literature review is more focused, the data sources are more transparent, the methodology is better explained, and the interpretation of PMC scores has been strengthened.

A few minor issues remain. First, the completeness and adequacy of the supplementary materials should be verified. Second, the discussion of robustness and sensitivity remains conceptual and does not include an empirical sensitivity test. Third, if Danesh et al. (2021) was used as a methodological reporting guide, the full citation should be added to the manuscript and reference list. Fourth, the Discussion section could still be slightly strengthened by adding a more critical comparison with previous studies.

Overall, the manuscript has responded well to the previous reviewers’ comments, and only minor revisions remain necessary.
