# Supplementary material for: Structural consistency in AI governance: A PMC index assessment with evidence from China’s central-level policies
Source: PLoS One. 2026 Jun 5;21(6):e0337024. doi: 10.1371/journal.pone.0337024 (PMC13240918; doi:10.1371/journal.pone.0337024)
Supplement: Attachment — Submitted filename: Response to Reviewers2.docx [file pone.0337024.s002.docx]

**Response to Reviewers #2**

（PONE-D-25-58681：Structural Consistency in AI Governance: A PMC Index Assessment with Evidence from China’s Central-Level Policies）

Thank you for your valuable comments and suggestions, which have greatly helped us improve our manuscript. We have carefully addressed each point and revised the paper accordingly. Below is our detailed response to your feedback:

1. **“*In the Introduction, the authors need to engage more deeply with the concept of artificial intelligence governance, clearly defining and theoretically framing this concept.”***
2. **“*The Introduction should not include methodological details. All content related to the research methods should be removed from this section.”***
3. **“*There is no need to describe the structure or sections of the paper in the Introduction, as this unnecessarily increases the length of the manuscript.”***

Thank you for these important comments. We agree that the Introduction should more clearly foreground the conceptual framing of artificial intelligence governance, while avoiding excessive methodological detail and unnecessary description of the paper structure. In response, we substantially revised the Introduction in the following two respects:

(1) We strengthened the conceptual and theoretical framing of the study by adding a clearer definition of artificial intelligence governance. In the revised Introduction, artificial intelligence governance is defined as the institutional, strategic, and regulatory arrangements through which the state guides, supports, coordinates, and constrains the development and application of AI. We also clarified why the structural consistency of policy design can serve as an analytical entry point for examining AI governance.

(2)We streamlined the Introduction by removing detailed methodological descriptions and the section-by-section outline of the paper. As a result, the revised Introduction now focuses more directly on the research background, the governance-related problem setting, the theoretical relevance of AI governance, and the overall research objective.

4. **“*The Literature Review section is overly extensive and should be substantially condensed. It should focus primarily on the core topic of AI governance and the application of the PMC index. The concluding synthesis of this section should also be more concise and focused.”***

Thank you for this helpful comment. We agree that the original Literature Review was overly extensive and that it should be more tightly focused on the core themes of artificial intelligence governance and the PMC Index. In response, we revised this section in the following two respects:

(1) We substantially condensed the review of AI policy studies and reorganized it around the core theme of artificial intelligence governance. In the revised manuscript, this section now focuses more directly on the evolution of AI governance research, the main approaches used in existing AI policy studies, and the key limitation of prior work—namely, its emphasis on policy themes, discourse, and instruments rather than on the structural consistency of policy systems.

(2) We also condensed the review of the PMC Index Model by emphasizing its methodological relevance to structured policy evaluation, rather than providing an overly detailed listing of applications across many policy fields. In addition, we substantially shortened and sharpened the concluding synthesis of the Literature Review so that it now focuses more clearly on the specific research gap addressed by this study.

5. **“*The data sources must be reported clearly and transparently, specifying exactly which databases or websites were used to collect the data.”***

Thank you for this helpful comment. We agree that the data sources should be reported more explicitly and transparently. In response, we revised the manuscript in the following two respects:

(1) In Section 3.3 Data Sources, we clarified the exact sources used to collect the policy documents. The revised manuscript now explicitly specifies that the corpus was retrieved primarily from the Chinese Government Website (www.gov.cn) and PKUlaw (pkulaw.com), and also reports the data retrieval period and the main screening principles applied during corpus construction.

(2) To further enhance transparency, we provide the full list of the 54 central-level AI policy documents as S1 File, so that readers can directly verify the composition of the policy corpus used in this study.

We sincerely appreciate this constructive feedback, as it has helped us improve the transparency and traceability of the study by making the policy data sources and corpus construction process more explicit.

6. **“*The methodology is not described with sufficient clarity. It is unclear which parts of the texts were used as input for text mining, what types of text-mining analyses were conducted, the sequential steps of the analysis, and which tools and techniques were employed.”***

**7.“*To improve transparency and rigor in reporting the text-mining methodology, the authors are encouraged to refer to the following study as a methodological guide:Danesh F, Dastani M, Ghorbani M. Retrospective and prospective approaches of coronavirus publications in the last half-century: a Latent Dirichlet Allocation analysis. Library Hi Tech. 2021;39(3):855–872.”***

Thank you for these important comments. We agree that the methodological workflow, especially the text-mining component and its relation to the PMC coding process, should be described more explicitly. In response, we revised the Methods section in the following three respects.

(1) We substantially revised Sections 3.2 and 3.4.1 to clarify the methodological sequence of the study. The revised manuscript now explicitly states that the full texts of the 54 policy documents were used as the input for text mining, and that the text-mining stage consisted of corpus preparation, Chinese word segmentation, word-frequency analysis, and semantic co-occurrence analysis using ROSTCM6.0 and NetDraw. We also clarified that these procedures were used to identify thematic features of the corpus and support variable construction, rather than to generate final PMC scores automatically.

(2) We revised Section 3.4.2 to explain more clearly how Tables 2, 3, and 4 were constructed. In the revised text, we specify that the indicator system in Table 2 was developed through the combination of prior policy-evaluation studies, corpus-based text analysis, and researcher interpretation of China’s AI policy texts. Table 3 is now described as the operationalized multi-input–output structure derived from Table 2, and Table 4 is explicitly presented as the PMC classification standard adopted from prior research.

(3) We further clarified that the study does not rely solely on automated text-mining analysis. Rather, it combines automated corpus processing with researcher-led interpretation and binary coding based on a predefined scoring matrix. To improve transparency in reporting the text-mining workflow, we also referred to the methodological reporting logic of Danesh et al. (2021) as a guide when revising this part of the manuscript, particularly in specifying the input corpus, preprocessing procedures, analytical sequence, and the relationship between automated analysis and researcher-led interpretation. In addition, the relevant supporting materials are now provided in the supplementary files.We sincerely appreciate this constructive feedback, as it has helped us strengthen the methodological transparency, interpretive clarity, and reproducibility of the study.

***8.“It is unclear whether the study relies solely on automated text-mining analyses or whether domain experts were involved in interpreting the results. For example, the analytical basis for Tables 2, 3, and 4 is not explained. This process must be described explicitly in the Methods section.”***

Thank you for this important comment. We agree that the original manuscript did not explain with sufficient clarity the respective roles of automated text mining and researcher interpretation, nor did it make the analytical basis of Tables 2, 3, and 4 sufficiently explicit. In response, we revised the Methods section in a more systematic way, so that the analytical workflow and the basis of each key table are now clearly specified.

(1) In Section 3.2 Research Method, we clarified that the study does not rely solely on automated text-mining analysis. Instead, the methodological process is sequential: text mining was first used to explore the thematic and semantic features of the full policy corpus, and the results were then integrated with prior policy-evaluation research, corpus-based interpretation, and structured researcher judgment to construct the PMC analytical framework. We now explicitly state that text mining supported corpus exploration and variable identification, but did not automatically generate the final indicator system or PMC scores.

(2) In Section 3.4.1 Policy Text Mining and Word Frequency Analysis, we further specified that the input for text mining consisted of the full texts of the 54 central-level AI policy documents, rather than only titles or selected excerpts. We also clarified the analytical sequence of the text-mining stage, including corpus preparation, Chinese word segmentation, word-frequency analysis, and semantic co-occurrence analysis using ROSTCM6.0 and NetDraw. This revision makes the technical workflow of the text-mining component more transparent.

(3) In Section 3.4.2 Variable Classification and Parameter Identification, we explicitly explained that Table 2 is not a direct output of automated text mining. Rather, it is a researcher-constructed indicator system developed through the combination of corpus-based findings, prior policy-evaluation literature, and structured interpretation of China’s AI policy texts. We also clarified that the detailed coding basis for all secondary indicators is provided in the supplementary scoring matrix.

(4) In Section 3.4.3 Construction of the Multi-Input–Output Table, we clarified that Table 3 operationalizes the indicator system presented in Table 2 by reorganizing the primary and secondary variables into a standardized binary coding structure for PMC evaluation. This makes clear how the indicator system was translated into the coding framework used for subsequent calculation.

(5) In Section 3.4.4 Calculation of the PMC Index, we explained that Table 4 serves as the interpretive classification standard for PMC scores, adopted from prior studies, rather than as a direct output of text mining or automatic coding. In other words, Table 4 is used to interpret the calculated PMC results after coding and index computation have been completed.

Taken together, these revisions make explicit that the study combines automated corpus processing with structured researcher judgment within a document-analysis framework, rather than external expert participation, and that Tables 2, 3, and 4 represent three linked but distinct stages of the analytical process: indicator design, coding structure, and score interpretation. We sincerely appreciate this constructive feedback, as it has helped us make the methodological logic of the study more explicit and the analytical basis of the key tables much clearer.

***9.“The Discussion section requires substantial rewriting. It should focus on the principal findings of the study and critically compare them with results from relevant previous research, rather than providing descriptive or repetitive content.”***

Thank you for this important comment. We agree that the original Discussion section relied too heavily on descriptive summary and did not engage sufficiently with the principal findings of the study in relation to previous research. In response, we substantially rewrote Section 5.1 Discussion in the following two respects:

(1) We reorganized the Discussion around the principal findings of the study, focusing more explicitly on the structural features revealed by the PMC evaluation—namely, the relative stability of the developmental-governance core of China’s AI policy system, the structural weaknesses in temporal planning and policy hierarchy, and the heterogeneity of incentive arrangements and influence levels.

(2) We strengthened the critical dialogue between our findings and relevant previous research. In the revised manuscript, the Discussion now compares our results with prior studies on AI governance, AI policy discourse, national AI strategy, and PMC-based policy evaluation. This revision makes clearer what the present study confirms, refines, and adds to the existing literature, rather than simply restating the empirical results.

We sincerely appreciate this constructive feedback, as it has helped us sharpen the analytical focus of the Discussion and better position our findings within the broader literature on AI governance and policy evaluation.
