# Supplementary material for: Structural consistency in AI governance: A PMC index assessment with evidence from China’s central-level policies
Source: PLoS One. 2026 Jun 5;21(6):e0337024. doi: 10.1371/journal.pone.0337024 (PMC13240918; doi:10.1371/journal.pone.0337024)
Supplement: Attachment — Submitted filename: 02Response to Reviewers.docx [file pone.0337024.s004.docx]

**Response to Reviewers**

**Manuscript Title:**

**Structural Consistency in AI Governance: A PMC Index Assessment with Evidence from China’s Central-Level Policies**

**Response to Previous Reviewer #1**

**Comment 1**

**Evaluation Outcome**

“*This concern has been largely addressed. The revised manuscript provides clearer explanations of where the data were obtained, how the policy texts were processed, and how the indicators were operationalized. The addition of a transparency and reproducibility section strengthens the methodological reporting of the study. However, the completeness and adequacy of the supplementary materials should still be verified to ensure that they actually include the full policy corpus, the detailed scoring matrix, and the complete PMC results.*”

Thank you for this helpful evaluation. We agree that, beyond the revisions made in the main manuscript, the completeness and adequacy of the supplementary materials should also be explicitly verified. In response, we re-checked all supporting files and ensured that they correspond directly to the main stages of the analytical workflow. Specifically, S1 File provides the full list of the 54 central-level AI policy documents included in the corpus, including their issuance years; S2 File provides the text-processing notes and lexical adjustment record used in the word-segmentation stage; S3 File provides the detailed indicator scoring matrix for the PMC evaluation system; S4 File provides the full PMC results for the five selected representative policies; and S5 File provides the underlying frequency data used to generate Figure 4. In addition, the binary coding results, primary-variable scores, PMC indices, and PMC matrices for the five representative policies are reported in Tables 6–8 of the main manuscript. We also clarified in the revised Data Availability Statement that the yearly distribution shown in Figure 3 is based on the corpus information provided in S1 File. These revisions were intended to ensure that the supplementary materials fully support the transparency, traceability, and reproducibility of the study.

**Comment 3**

**Evaluation Outcome**

“*This concern has been partially addressed. The authors have discussed robustness and sensitivity at a conceptual level, which is acceptable given that the previous comment requested a brief discussion. However, the manuscript still does not include an empirical robustness test or sensitivity analysis. Therefore, this issue has been addressed at the discussion level, but not fully resolved empirically.*”

Thank you for this helpful evaluation. We agree that the current manuscript addresses robustness and potential sensitivity mainly at the conceptual discussion level rather than through an empirical robustness test. In light of the original review request, which asked for a brief discussion of robustness and sensitivity, we retained this discussion-oriented treatment but further clarified its scope in the revised manuscript. Specifically, we now state more explicitly that the robustness discussion is intended as a conceptual qualification rather than an empirical sensitivity analysis, and that the findings should therefore be interpreted as structurally informative rather than numerically absolute. We also emphasize that future research may examine robustness more systematically by testing alternative indicator configurations, different case-selection strategies, or broader policy samples.

**Response to Previous Reviewer #2**

**Comment 7**

**Evaluation Outcome**

“*This concern has been partially addressed. The reporting of the text-mining procedure has improved. However, a clear full citation to Danesh et al. (2021) was not identified in the revised manuscript or reference list. If the authors used this study as a methodological reporting guide, the full citation should be added to the text and reference list.*”

Thank you for this helpful evaluation. We agree that, if Danesh et al. (2021) is referred to as a methodological reporting guide in our previous response, the corresponding citation should also be clearly included in the revised manuscript and reference list. In response, we have now added an explicit citation to Danesh et al. (2021) in Section 3.2 Research Method to indicate that the reporting of the text-mining procedure was further refined with reference to prior methodological studies on transparent text-analytic workflows. We have also added the full bibliographic information to the reference list. This revision was intended to make the methodological basis of our reporting more explicit and consistent between the manuscript and the response letter.

**Comment 9**

**Evaluation Outcome**

*“This concern has been largely addressed. The revised Discussion section is more analytical and better organized around the main findings. It also links the findings more clearly to previous research. However, the critical comparison with prior studies could still be slightly strengthened*.”

Thank you for this helpful evaluation. We agree that the revised Discussion section could be further improved by making the comparison with prior studies more explicit. In response, we have slightly strengthened the concluding part of Section 5.1 Discussion. Specifically, the revised text now states more directly that, compared with prior studies focusing mainly on policy themes, discourse, or instrument types, the present study further highlights the structural coordination problem of China’s central-level AI policy system across time horizons, governance levels, and implementation tools. We also clarify that the study extends existing AI governance research by shifting the analytical focus from thematic description to structural consistency. These revisions were intended to make the comparative and critical contribution of the Discussion more explicit.
